# Commentary: First-Order Embodiment, Second-Order Embodiment, Third-Order Embodiment

**DOI:** 10.3389/fpsyg.2018.00445

**Published:** 2018-04-04

**Authors:** Lisa Quadt

**Affiliations:** Psychiatry, Department of Neuroscience, Brighton and Sussex Medical School (BSMS), University of Sussex, Brighton, United Kingdom

**Keywords:** embodiment, representation, predictive processing, grounding, free-energy principle

The framework of first-, second-, and third-order embodiment (1-3E; Metzinger, 2014) was developed for exploring how phenomenal properties are physically and computationally grounded. Metzinger's goal for 1-3E is to show how the experience of being a self (i.e., phenomenal selfhood) is generated within an embodied system and thus to embed his self-model theory (Metzinger, 2004) in the context of grounded cognition. The basic claim of his approach is that phenomenal selfhood (3E) is grounded in computational, representational processes (2E) that in turn are grounded in physical (i.e., neural and bodily) structures (1E). In what follows, I will discuss the aspects of grounding and representationality in the framework, and suggest clarifications.

In a hierarchical framework such as 1-3E, a necessary question is how different levels relate and connect to each other. Metzinger emphasizes that there are grounding relations holding between first-, second-, and third-order embodiment. He proposes Pezzulo's et al. (2013) conception of a grounding theory of cognition as a fitting theoretical framework, where grounding refers to a physical foundation. An example of this can be found in the original article, where Metzinger suggests that eye movements ground the phenomenal experience of lucid dreams (p. 276). In general, grounding refers to the relation between levels of embodiment that give an insight into how high-level properties, such as the experience of selfhood, emerge from low-level properties. Metzinger gives a description of the relation between 2E and 3E, where the representational *content* of 2E is “elevated to the level of global availability and integrated with a single spatial situation model plus a virtual window of presence” (p. 274). It is, however, less obvious how these two levels of embodiment relate to the lowest level 1E.^1^ 1E systems are described as “purely physical, reactive” (p. 273) systems that exploit their physical resources to navigate their environment. This is merely a description of a 1E *system*, and does not refer to how 1E can amount to a grounding level *within one* system that also possesses 2E and 3E (Quadt, 2017).

This problem also relates to the notion of representationality in the framework. Metzinger claims that in order to arrive at a characterization of the phenomenal properties of selfhood, they need to be described in terms of their representational properties. These representational properties then have to be “bottomed out” (p. 277) by formulating a computational model.

The final step then is to find necessary, enabling and constitutive parts of phenomenal selfhood. This will help finding grounding relations, for “grounding is about constitution.” (p. 277) Metzinger asks the question of how exactly the unconscious body model at 2E is grounded in 1E and suggests to start with describing representationality as a gradual property that allows the phenomenal self-model to “bottom out” into non-representational dynamics (p. 278). What is needed, it appears, is an account of grounded representational models that sheds light on the actual relations between 2E and 1E. I suggest that the framework of predictive processing (PP)—more specifically, its embodiment-focused version (e.g., Clark, 2016)—will offer valuable insights.

To begin with, consider the claim that representationality is a gradually arising process, and not an all-or-nothing phenomenon. This sits well with the core assumption of PP that there is a functional processing hierarchy with actual sensory input at the bottom of it (Palmer et al., 2015). At the next higher level, according to PP, already exists an abstraction of these inputs, namely a predictive, probabilistic model of the input. These abstractions can be called representations (as they represent a prediction of what the input at the level below is), and they are grounded in the actual signal in virtue of prediction errors carrying information about the external world, providing a “grip” on the environment. This notion of representation is quite flexible, allowing for the presence of inner models at every level of the hierarchy. Representationality then arises as a gradual phenomenon whose degree of abstraction increases. Applied to 1-3E, this means that representational processes at each level of embodiment can be differentiated by their degree of abstraction from the actual sensory input and by their degree of representationality. While 1E might display only little representationality, this increases as one goes up the hierarchy.

However, the question of how these gradually arising representations are grounded remains. PP is an instance of the free-energy principle (Friston, 2010), and it is here that we find a hint to an answer. The free-energy principle aims to explain how biological organisms maintain their integrity in an ever-changing environment. Friston and Stephan (2007) suggest that biological systems achieve this by embedding thermodynamic laws into their anatomy. This means that the physiology of a system constrains their living circumstances—a fish needs to live in water, a cat must live on land given their phenotypical anatomy. This is what Friston (2011) calls “embodied inference”—instead of systems merely *having* or *computing* (representational) models, embodied systems *are* models of their environment. More specifically, their morphology incorporates (*models*) the laws of its surroundings to ensure its survival—an organism's phenotype determines its possible state space. An agent's body thusly enables and constrains its sensorimotor trajectory. Whether or not, for example, an individual needs to stay in salty waters to ensure survival necessarily determines its adaptive behavior. An agent's morphology sets constraints that dictate further processing—in the context of PP, we may speak of bodily priors. A higher-level body model therefore must represent an agent's actual morphology in relation to other objects or individuals and its possible sensorimotor trajectory. The answer to the question of what grounds the body model could thus be: The embodied agent who is a model of its environment. Moreover, the concept of “morphological computation” (Pfeifer and Gómez, 2009) provides insights in how morphological features of an organism simplify and facilitate complex motor, locomotor, and sensory tasks. By “taking over” part of the computational load, the organism sets up constraints for higher-level representational processing.

How then can we characterize the relationship between levels of embodiment in the 1-3E framework? Taking into account PP and the free-energy principle, one possibility is that 1E profoundly constrains and shapes 2E. The physical structure of a biological organism is described at the level of 1E and determines what is and *can be* represented at 2E. The structural elements may not explicitly be represented at the higher level, but they seem to constrain and influence the structure of the representational body model at 2E. If we assume that 2E amounts to a virtual, predictive body model that integrates multimodal inputs (p. 273), then 1E sets up its basic structure (Quadt, 2017).

## Author contributions

The author confirms being the sole contributor of this work and approved it for publication.

### Conflict of interest statement

The author declares that the research was conducted in the absence of any commercial or financial relationships that could be construed as a potential conflict of interest.

## Footnotes

^1^The reason for this shortcoming may be his focus of attention, as he clarifies in a reply to Gallagher (Metzinger, 2006); his main interest lies in the relation of 2E and 3E.
